# Characterization of People with Functional Limitations from ICF Components Using the Longitudinal Social Protection Survey (ELPS) of Uruguay

**DOI:** 10.3390/ijerph18158012

**Published:** 2021-07-29

**Authors:** Julia Córdoba, María José Bagnato

**Affiliations:** AD-Instituto de Fundamentos y Métodos en Psicología, Facultad de Psicología, Universidad de la República (UdelaR), 11200 Montevideo, Uruguay

**Keywords:** disabilities, health care, public health, dependency

## Abstract

Characterising people with disabilities at the population level using the ICF approach is a challenge, as it implies that researchers are able to identify variables that can account for the components that make up the multidimensional definition of disability. The purpose of this study is to generate updated information on disability in Uruguay, as there has been no in-depth analysis of how this population lives, how they access the services and benefits that affect their quality of life, and what the significant differences are between those who make up this population. A quantitative analysis was applied to the target population, consisting of participants in the Longitudinal Survey of Social Protection (2016) who reported at least one limitation in performing ADLs and who were in the age range of 18–64 years. Significant differences were found between the different groups in terms of their reported limitations in relation to obtaining necessary services due to their health condition, dropping out of education before completing the compulsory level, low labour market insertion, feelings of loneliness, and low participation. More research needs to be done as it is clear that people with disabilities do not have access to the support they need, which leads to even greater exclusion.

## 1. Introduction

The approach to disability proposed by the World Health Organisation, through the International Classification of Functioning, Disability and Health [1], allows us to understand this issue, accounting for its complexity and multidimensionality. In this model, disability is postulated as a complex and dynamic concept and is defined as the negative result of the interaction of a health condition, the limitations it generates in daily life activities, and the barriers and facilitators that a person may have in their environment that restrict their participation. The ICF makes it possible to understand and study disability through a biopsychosocial approach to health based on three components: the person’s health condition (body structures and functions), their daily life activities, and their social participation. The first component, health condition, is necessary in the consideration of disability status, although it is not sufficient on its own. It is related to the effects of the body’s functions and structures in terms of the person’s performance in the realisation of the activities of daily living (ADLs) and the restrictions on the person’s ability to participate in social activities. Finally, it incorporates contextual factors (environmental and personal) that allow the analysis of the presence (facilitators) or lack (barriers) of resources that the person can count on [1].

In situations of disability, so-called health disparities [2] arise, which are significant differences in relation to health issues, both clinical and statistical, when comparing populations with and without disabilities. Being able to understand these inequalities in the disabled population is important at a global level because, as numerous studies [3,4,5,6,7] have shown, this group is not homogeneous either.

This article analyses disability based on health status and factors from a population-based survey, the Longitudinal Social Protection Survey (ELPS), in Uruguay. The study is based on a comparison between participants who reported having at least one functional limitation.

The health of people with disabilities is context-dependent [8] and therefore cannot be reduced to the diagnosis of a health condition and its treatment and rehabilitation. This can be a determining factor when planning clinical interventions, as well as in the planning associated with government programmes and services that aim to improve the quality of life of people with disabilities and their families [9]. The production of information and the characterisation of these populations should consider other aspects in addition to their number, such as, for example, their support needs (human, animal, technical, and/or technological), the benefits they have access to and those that have been refused, their health care beyond that associated with their specific condition, their educational and employment trajectory, and their social inclusion. These studies must be contextualised according to the life cycle, as there are differences in people’s needs at different stages of life [10].

Some examples of the suggested type of study are those that analyse disability and socioeconomic status [11,12]; disability, mortality, and morbidity [4,13]; comparisons between types of disability [5]; of the differences that may exist in people with the same type of disability [6,10,14]; of the different disabilities that the same health condition may generate [15]; and studies of the prevalence of types of disability depending on whether one lives in a rural, urban, or suburban area [16].

Analysing specific dimensions of the population with disabilities, such as their health, through censuses and surveys is challenging due to the different operationalisations of the concept and, therefore, the modules, questions, and variables designed and used in each study. The working-age adult population suffers especially from the lack of agreed definitions, which generates fragmented and non-specific information on the health status of this group [17]. The WHO has facilitated this process through the generation of specific survey modules in censuses and surveys [18], as well as the definition proposed by the ICF.

Tamayo et al. [19] propose that disability is a social determinant of health, since the existence of a health condition, limitations in ADLs, and/or restrictions in social participation impact the possibility of accessing services and benefits that could compensate for these difficulties. If those who need these supports do not access them, a cycle of exclusion begins, therefore resulting in socio-economic vulnerability, which perpetuates and deepens the situation of disability [20]. So-called unmet needs arise, which are those supports that people claim to need but have not received through benefits and/or programmes [21]. It becomes necessary to develop robust and consistent methods that produce information on the health of working-age adults with disabilities [17].

Even considering the advances in the unification of criteria, as in the case of the ICF, and the operationalisation of these variables in population-based surveys, such as the aforementioned work of the WG, the production of multidimensional information on disability continues to be scarce [8]. Information on the population with chronic health conditions that may evolve into future situations of disability is also scarce [22]. Uruguay has an important legal framework to support people with disabilities. The Law on the Comprehensive Protection of Persons with Disabilities [23] is based on the Convention on the Rights of Persons with Disabilities [24] and aims to guarantee access to specific benefits and services for this population to ensure equal opportunities. With regard to specific access to health care, the National Integrated Health System (SNIS) [25] ensures health coverage for the entire Uruguayan population, whether through a public or private provider. Although the SNIS requires certain goals for the provision of health services, there may be differences in terms of the type and quality of the services provided. Finally, there is the National Integrated Care System (SNIC) [26], which regulates services and benefits for people in a situation of dependency; among these are people who are dependent due to disability. As in the SNIS, within the framework of the SNIC there are public and private providers, the services of which include, for example, personal assistance, day centres, long-stay centres, and telecare.

Three nationally representative population-based surveys have been carried out with aspects relating to disability: the National Survey of Persons with Disabilities [27], the Housing and Household Census [28], and the Longitudinal Survey of Social Protection [29]. These three studies used the recommendations of the WG, which allows for international analysis and the comparison of the situation in Uruguay with those in other regions and/or countries.

The variety in the sources of information, the need to deepen the analysis of the subject, and the internationally agreed-upon definitions of disability motivated this study, which aims to examine the relations between impairments considered to be health conditions and functional limitations, and to explore their relationship with their context, based on the perceived need for support and the benefits obtained.

The aim of this study was to characterise the situation of people who reported having a functional limitation in the second wave of the Longitudinal Social Protection Survey (BPS, 2015) according to the components of the ICF. The specific objectives were (a) to determine the health disparities between the different types of disability through the data collected in the ELPS; (b) to identify the unmet needs declared; and (c) to analyse, through the definition of barriers and facilitators, the information organised according to the contextual factors of the ICF.

## 2. Materials and Methods

### 2.1. Data and Variables

Information was obtained from the Longitudinal Survey of Social Protection [29], which is a longitudinal study, representative of the Uruguayan population. Two waves have been conducted to date, with a response rate of 14,674 people. In this study, we used the information collected during the second wave, in 2016, as if it were a cross-sectional survey. The population o selected for analysis consisted of people between 18 and 64 years old, divided into 5 groups (18–29, 30–39, 40–49, 50–59, and 60–64) who reported having at least one limitation in performing activities of daily living; both of which are variables surveyed in the ELPS. Based on the theoretical framework of the ICF, the criterion of disability status was assigned to those who indicated having at least one functional limitation.

For the analysis of the data, a grouping of the originally surveyed limitations was made and 6 groups were determined: those with “more than two limitations” and people who reported having only “limitations in seeing, even when wearing glasses”, “limitations in communication and speech”, “physical limitations”, “limitations in learning and understanding”, and “limitations in relating to others”.

Socio-demographic differences between these groups of limitations were analysed, including age, gender, educational level, working status, and residential zone (capital city or inland). Finally, the life situation of each of these limitation groups was studied through the contextual factors of the ICF. For this purpose, among the ELPS variables, those that could account for both personal and environmental factors were selected. Table 1 provides the definitions of each of the factors and details this operationalisation process.

The operationalisation of the contextual factors from the ELPS variables requires the description of each of the latter. Some of them can be grouped into categories of questions designed to survey a broad topic, for example, the question of limitations for ADLs or the need for help, and others are more specific and only require one category to be surveyed, such as the case of whether a mammography examination was performed in the last 12 months.

*Limitations in performing ADLs.* This question identifies individuals who report having a limitation in performing ADLs and is based on the WG Long Set [18]. In addition to identifying the prevalence of limitations, this study identifies whether this limitation affects daily life.

*Origin and cause of health condition*. This study considers the declared limitation considered to be the origin (mental, sensory, and physical) of the health condition that affects an organ, structure, and/or function; this variable was operationalised in the groups of limitations mentioned previously. Analysing the cause of these difficulties based on the ELPS, and on any self-reported survey, has its limitations, since these are responses constructed from the particularities of the person’s history in relation to their situation. Their account is associated, for example, with the time of occurrence of these difficulties or whether they had access to medical information about what was happening to them or whether it was based on a family account [30].

*Need for help to perform ADLs.* This question is based on the definition of the National Integrated System of Care [25]. Based on this reference, this module was constructed in the ELPS, which surveys the need for help from a third person to carry out ADLs (basic, instrumental, advanced). In addition to the prevalence of the need for help, it also shows who, among these persons, require maximum substitution; that is to say, they need all their activities to be carried out by another person.

*Need for support.* In line with the need for help, this group of questions identifies not only the need for support on the basis of the declared limitation, but also describes whether or not they receive it. Supports are understood as a need in a given situation, based on the particular person [10,31]. No reference is made to the CRPD definition [26] as it is understood that the ELPS does not reach the complexity of the convention in terms of accessibility and universal design.

*Highest level of education.* Due to the fact that the ELPS has more than 15 options to describe the educational level reached by the participant, this study proposes re-categorising the variable “highest level of education attained” by associating it with the definition of compulsory education according to Uruguayan regulations.

*Diagnosed illnesses.* The survey categorises 18 illnesses and allows for the entry of a diagnosis not included in the “other” category. In this study, by categorising the responses obtained in the “other” option, 11 diseases were included, resulting in the following list: asthma, emphysema, atrosis/arthritis, tendinitis, rheumatism, hypertension, diabetes, osteoporosis, kidney failure, heart problems, spinal problems, anaemia, cancer, digestive/hepatic problems, neurological problems, mental health disorders, thyroid problems, Down syndrome, ASD, Parkinson’s, dementia/Alzheimer’s, autoimmune diseases, epilepsy, fibromyalgia, obesity, alcoholism, celiac disease, allergies, and HIV-AIDS.

*Orthoses and prostheses*. Examples used in the ELPS to define orthoses are “spectacles”, “hearing aids”, “crutches”, “wheelchair”, and “walkers”. Prostheses refer to artificial structures that replace a bodily structure due to its absence.

*Contextual factors not considered.* According to the ICF categories, there remained four contextual factors to which no survey variables were assigned. These were “major life areas”, “general tasks and demands”, “natural environment and changes in the environment due to human activity”, and “attitudes”.

### 2.2. Statistical Analysis

All analyses were conducted using the Statistical Package for the Social Sciences, version 22 (SPSS 22.0) (IBM, Chicago, IL, USA). Descriptive analyses were conducted on sociodemographic characteristics and the variables assigned to each factor, described in Table 1. The prevalence of the variables was estimated by the whole sample used in the study and by each of the groups of limitations, considering them as the dependent variables. Student’s *t*-test was used for independent samples and significance levels were set at 5%.

## 3. Results

The results are presented in three subchapters. The first one describes the socio-demographic data and aims to present the general characteristics of the target population. The second and third subchapters respond to the categorisation of the ICF contextual factors and personal and environmental factors. Thus, in these subchapters the ELPS results are analysed according to the variables associated with each factor mentioned.

The sample comprised a total of 1032 people, including 374 males (36.2%) and 658 females (63.8%) with a mean age of 48.7 years (range 18 to 64). Table 2 describes the sociodemographic characteristics of the study sample.

According to the data collected in Table 2, it is important to be able to determine the moment of occurrence of the limitation that most affects the person’s daily life and restricts his or her participation, in this case in the educational and work environment. In spite of this, more than 60% of those who only reported learning difficulties had reached the highest level of primary education, a compulsory level of education in Uruguay. Less than 10% completed secondary school and no cases were reported of those who had completed higher education.

In relation to employment, almost 90% of those with relationship limitations had not worked for at least a week and 50% stated that they had not been looking for a job either. Almost half of those with physical limitations were not available for work and the same is true for almost 25% of those with limitations in seeing and communicating and speaking. For those with more than one limitation, more than 65% neither worked nor looked for work in the previous week.

As it can be seen in Table 3 of the total study sample, only 211 people (20.4%) reported a diagnosed illness out of a total, as mentioned above, of 29 health conditions categorised. Among the diagnoses reported were cardiovascular diseases, cancer, and respiratory problems, which cause 63% of global deaths. In lower income countries they account for 80% of deaths in the age group of the target population of this study [32].

In three types of diagnoses, the participants declared that their health condition affected more than two limitations: 40.7% of those with celiac disease, 50.0% of those with neurological diseases, and 100% of those with Down syndrome. Although mental health disorders were a possible response option for the participants, there were no cases in which they were declared. It is interesting that people who responded that they only had difficulties in relating to others did not have a diagnosis of mental health issues.

The assignment of the ELPS variables to each factor, as presented in Table A1, allows us to describe them and to identify which dimensions act as barriers and which as facilitators.

### 3.1. Personal Factors

#### 3.1.1. Learning and Application of Knowledge

Excluding the category that includes people with only learning difficulties, the only grouping that shows limitations in this area is the one with more than one limitation; in both groups, more than 80% of people reported that it affected them in everyday life. Despite this, more than half of those requesting support for this difficulty did not receive it. For these two categories, more than one limitation and learning disabilities, the percentages of illiteracy were between 20.5% and 23.7%, whereas the illiteracy rate of the country for the year in which the sample was applied was 1.4%.

The percentages in relation to the completion of compulsory education become more homogeneous between the groupings but only with statistical significance for the case of “more than one limitation” and “limitations for seeing”. In both cases, more than a fifth of the respondents did not complete the compulsory level (primary and lower secondary), in line with the population statistics at the time of Wave 2 [33].

In the case of those with specific learning difficulties, as seen above, a significant percentage did not complete compulsory education. Although it is clear that specific needs in this dimension may not only require support linked to cognitive processes, the need for support and/or adjustments is evident, so that the impact in the area where this limitation develops can be diminished.

#### 3.1.2. Communication

One of the aspects to highlight in the results obtained is the relationship between limitations and the need for help, in this case from a third person, that is, the concept of dependence [34]. It should be noted that the variable “need for help to communicate and make decisions” includes, in addition to the communication aspect, decision making, which may explain why some respondents who stated that they did not have communication limitations reported that they needed help to make decisions.

There is a significant difference in this aspect between the two categories that reported having limitations and needing help with communication, more than one limitations, and limitations in communicating and speaking. As can be seen, the need for the maximum type of help in the case of the first category can be explained by the operational definition of the category, as it was built on the premise of having more than two health conditions. If we analyse the category that only presents difficulties in the area of communication, the need for help from other people to communicate is not of maximum substitution.

#### 3.1.3. Mobility

More than 90% of people with physical limitations, whether in the movement of hands, arms, legs, and/or feet, stated that it affected their daily life; even so, more than half of them did not receive support for the adaptation of their home to facilitate their mobility.

For both the “more than one limitation” and “physical limitations” categories, the need for help that was most required was for mobility of the body position and not for mobility within the home. Again, it is not maximum substitution that is required from the other person, but more subtle and ad hoc assistance.

Being the two largest groups in the sample in absolute terms and those who reported the greatest effect on daily life activities, less than 15% required support and, of these, half did not receive it.

#### 3.1.4. Self-Care

The variables that describe this factor are associated with the need for help from another person, dependency, and, on the other hand, three compulsory check-ups for those residing in Uruguay: Pap smear testing, mammography, and prostate examination. We also asked whether they had consulted a doctor in the previous 12 months. In this way, it is possible to understand the factor in self-care linked to the protection of oneself and personal hygiene, as well as health care.

The two categories that had the greatest impact on the performance and/or capacity of this factor were “more than one limitation” and “physical limitations”, but there were clear significant differences. In the first case, the need for self-care was greater for tasks related to applying medical measures, avoiding risk situations, and asking for help, whereas the opposite was true in the case of physical limitations, where the need for help was associated with personal hygiene tasks and to a lesser extent with risk prevention and health maintenance.

The reason that the “more than one limitation” category showed the highest percentage of consultations with a doctor in the last 12 months is perhaps because this group has several health aspects to attend to.

Relationship, understanding, and learning and physical limitations were the categories in which most people reported not having had a Pap test or mammography in the previous year. Both are routine in Uruguay, the former from the age of 21 and the latter from the age of 50. A similar situation occurs when analysing prostate screening; more than 60% of people who should have had the test did not do so.

#### 3.1.5. Domestic Life

This dimension differentiates between moving around the home as a task linked to understanding the uses of places and the possibility of moving between them and, on the other hand, the tasks that need to be carried out to maintain the home. In this sense, people with physical limitations, those who had difficulties in the incorporation and planning of tasks, and those who presented comorbidity declared that they needed support to be able to carry out this task (A-ADL; composed of I-ADL).

It is in this factor where one can observe, for the first time, a high number of people needing total help to carry out the task, as well as the prevalence of people needing support to carry it out, almost a third of whom did not receive it. That is to say, this was one of the factors in which the greatest amount of help was required and the least amount of support was received.

#### 3.1.6. Interactions and Interpersonal Relationships

The only variable found that could contribute directly to this factor was that of limitations in relating to other people, which means that the factor was closely linked to mental health. As can be seen in the table of results, those who responded to this factor were in the “more than one limitation” and “relationship limitations” categories.

Notably, more than 90% of people reported that their daily life was affected by this health condition.

#### 3.1.7. Community, Social, and Civic Life

The construction of this factor was carried out, considering that people should not only be able to participate on equal terms with others but, at the same time, they should be able to access and/or arrive at spaces for social encounters.

The difference that emerges is that members of the categories “limitations in communicating and speaking”, “limitations in understanding and learning” and “limitations in relating” showed, in absolute terms, a greater need for help in order to take part in instances of participation. On the other hand, “more than one limitation”, “limitations in seeing” and “physical limitations” showed a greater need for help to move around outside the home, which could condition real participation in collective instances.

### 3.2. Environmental Factors

#### 3.2.1. Products and Technologies

This factor has two types of variables: those associated with specific products that can compensate for a deficit of function or an absence of structure, and those describing access to publicly available technological products.

More than a third of respondents, in absolute terms, in all categories except “relationship limitations”, did not know how to use a technological device. These included causes of limitations that, with technological resources, e.g., for content adaptation and technical adjustments (hardware and/or software), could facilitate the ability to perform I-ADL and A-ADL.

#### 3.2.2. Support and Relationships

This factor is broken down into three variables relating to (1) support from another person, (2) whether respondents are interested in a care programme that can provide that support, and (3) whether they feel lonely.

In relation to this last variable, 75.0% of those who had difficulties with relationships did feel lonely, but despite this they were not interested in benefits or support. In relation to the feeling of loneliness, in second and third place came the groupings of “limitations in learning and understanding” with 59.3% and of “more than one limitation” with 46.7%. Of the latter group, 65.3% were not interested in a care programme or did not know about it.

The difference in the data on help from another person received by people with more than one limitation (61.3%) compared to those with only visual limitations (9.7%) is partly due to the fact that people with visual limitations are not dependent upon others. In both cases, the vast majority of those who received this help received it from an unpaid carer; the ELPS in this case provides three options: a member of the household, a relative from another household, or a non-relative.

#### 3.2.3. Services, Systems, and Policies

In all categories, participants said that they had been rejected from receiving benefits that specifically impacted their health condition. Similarly, all the respondents had received temporary subsidies for aggravations of their condition and food support services. In relation to the financial support they said they needed, more than 60% did not receive it.

## 4. Discussion

Each limitation category contained factors in which variability in performance, ability, limitation, and functioning could be observed. For the ICF, functioning is the positive version of the interaction of these categories with contextual factors, and disability is the negative version [1]. The structure that the ICF provides through its components facilitates this type of analysis as it not only disaggregates them to be studied in detail but also allows researchers to understand their interaction.

The results showed that although there may be a deficit that affects, for example, communication and speech, this does not mean that it affects them on a daily basis or that it requires support or adjustment to compensate for it [5,6,8,17,35].

The ICF suggests a concept, the “health-related domain” [1] (p. 220), that encompasses those aspects that are not within the medical scope of health but that have a health-related impact on the quality of life of the individual; and it is this approach that allows us to identify the presence of health disparities among people with certain health conditions.

A relevant example is that of Pap smears and mammography. The lack of resources to facilitate and adapt the procedure for people who have reduced mobility or find it difficult to understand and accept the examination that the test requires [36] may explain the results of this study.

Medical care generally focuses on aspects related to the respondents’ health condition and does not examine the suggestions and implications of the non-specific epidemiology of people with disabilities. Due to lack of access to physical exercise and attention to the potential risk factors associated with cultural practices and unhealthy habits, people with disabilities tend to exhibit comorbidities [9,37], which tend to increase with increasing age [35] and thus aggravate the situation of disability. Having health coverage or up-to-date medical check-ups is also not synonymous with access to health, as seen in the “more than one limitation” category. Having medical consultations and check-ups does not guarantee that the person is accessing the support and adjustments required to be able to function in daily life on an equal footing with others [22,38,39].

Mental health issues are an example of this situation, as it is a difficult subject to address socially, which may generate three types of non-exclusive problems: difficulty in accessing diagnoses, stigma associated with mental health disorders may be causing under-reporting, and, finally, limitations may be caused by aspects not linked to health conditions but resulting from socio-economic situations. The latter may be part of what was defined above as a cycle of exclusion. The “interactions and interpersonal relationships” factor is linked in this study only to aspects of mental health, which does not collaborate with the social dimension of the ICF approach, since the description of the factor is associated with whether respondents have difficulties in relating to others. The lack of information required to identify and diagnose this type of condition; to assess their impact at the personal, family and community level; and to consult experts in the field is a public health problem worldwide [8,40,41,42].

This reality becomes more complex when taking into account the phenomenon known as the disability paradox, which is the difference between the objectifiable difficulties generated by certain health conditions and the quality of life declared by the person living with them [43]. The disability paradox is reflected in other dimensions analysed in this study, for example, the need to be helped, whether by other people, technical devices or programmes and services. Although people reported that they needed some assistance in everyday life, they did not report that state programmes would be able to help them. Again, this may be due to a lack of information about these services, their own health condition, and/or the stigma associated with the latter [8,9,10].

The support that this population receives is more associated with their situation of socio-economic vulnerability, such as receiving food aid, rather than with their condition. In other words, they do not have access to specific forms of assistance that could compensate for certain difficulties or help them to carry out everyday tasks. They do have access, and to a lesser extent than requested, to benefits that aim to respond to needs generated by the lack of access to education and employment. This means that people are subject to these benefits, which are generally governmental, and to the impossibility of what their own capacity and performance could produce [44,45].

The supports that do reach the population and that are linked to their health condition could benefit from flexibility in administrative and management definitions; an example of these possible adaptations is that the allocation requirement could involve a certain profile of limitations and the need for support, rather than not categorical definitions such as “severe”, “moderate”, or that the provision of aid could be adjusted according to the frequency and daily rhythm of the person and their family.

In order to promote and guarantee the inclusion and autonomy of people with disabilities and/or dependency, it is essential that these supports are adjusted to the specific needs of recipients. Examples of this are the results in relation to the work trajectory of this population, of which a significant majority had not worked in the previous week and were not available to do so [46,47]. On the other hand, it is important to study how much people are aware of the existence of these supports, and there could be an under-reporting of the need for this type of help to compensate for limitations. An example of this is that more than a third of the people who responded that they had more than one limitation were not aware of the existing care programmes.

The benefits and programmes that this population may be receiving do not consider the life trajectory of the person or their impact in the future. That is to say, there may be occasional support at specific moments in the life of a person with a disability, but once he or she no longer meets the profile, for example, age or area of inclusion, the person is left without support. The support provided by the education system must be designed so that the person can incorporate their learning into their participation in the labour market and labour inclusion policies must take into account the fact that many of the people who make up this population may not meet the requirements demanded of the wider population due to a disparity in access to training [35,48,49].

Participation, through the “community, social, and civic life” factor, is a clear example of how it is necessary to contemplate specific situations, not individual ones, and to generate profiles of need that make it possible to contemplate the multiple limitations and, therefore, multiple life situations that a health condition can generate [49].

## 5. Conclusions

This study concludes that the ICF is a good conceptual framework and technical tool at the population level for the study, description, and international comparison of disabled populations. None of its components has a linear or univocal relationship with the other components, corresponding to the empirical multidimensionality of the subject. Reality shows that benefits and programmes can be installed as facilitators for some people when they access them, or as barriers when they are rejected and hinder access to other spaces, whether for the purpose of participation, employment, education, or leisure.

Uruguay is a country with a broad legislative framework aimed at improving the quality of life of people with disabilities; however, there are large differences between what these regulations establish and what actually reaches people with disabilities. It is urgent to take action with regard to this population, especially for people with multimorbidity or multidisability, as they are the ones who require the most support and who are receiving the least, as can be seen from the results presented in this study. Research should be conducted on what type of support is needed, contemplating designs that can be adjusted to the needs of each situation with the objective of promoting autonomy and social inclusion.

It is therefore essential to continue with this type of characterisation in order to further deepen the dimensions that are critical to guarantee the improvement of the quality of life of this population and their families.

Although the ELPS is a survey that allows for a detailed characterisation of the situation of people with disabilities, it is not a specific survey on the subject, so it is necessary to highlight at least two limitations that may be related. The data were obtained via self-declaration, and the data collection teams, although they were trained for this task, were not technicians with specific expertise in the assessment of disability. This may lead to both the under-reporting and over-reporting of information.

## 6. Ethical Considerations

The Banco de Previsión Social, the institution that generated the data, made the database anonymised and available for analysis by research teams upon request. 

## Figures and Tables

**Table 1:** Correspondence of contextual factors (CIF) with analysed ELPS variables.

Contextual Factor Classifications	ELPS Variables
1. Personal Factors	
1.1 Learning and Application of Knowledge	
Refers to learning, the application of learned knowledge, thinking, problem solving, and decision making.	Understanding and learning limitations, cannot read and write, needs support for learning, completion of compulsory education
1.2 Communication	
Concerns general and specific aspects of communication through language, signs, or symbols, including receiving and producing messages, conducting conversations, and using communication tools and techniques.	Communication and speech limitations, need for help with communication and decision making
1.3 Mobility	
Refers to movement when changing body position or location; when picking up, moving, or manipulating objects; when walking, running, or climbing; and when using various means of transportation.	Limitations in the use of the upper limbs (arms and hands), limitations in the use of the lower limbs (legs and feet), needs assistance to change and maintain body position, needs assistance to move around in the home, needs assistance to adapt living arrangements
1.4 Self-Care	
Deals with personal care, understood as washing and drying oneself, care of the body and body parts, dressing, eating and drinking, and taking care of one’s health.	Needs help to avoid health risks, ask for help, or follow treatment; needs help to get dressed; needs help to comb hair, cut nails, brush hair, or clean teeth; did not have a Pap smear; did not have a mammogram; did not have a prostate check-up; did not have a medical consultation in the last 12 months; had a medical consultation in the last 12 months
1.5 Domestic Life	
This category details how to carry out domestic and everyday tasks and actions. Areas of domestic life include obtaining a place to live; obtaining food, clothing, and other necessities; cleaning and repairing the home; caring for personal and household items; and helping other people.	Needs support for daily tasks Needs help with cooking, shopping, cleaning, etc.
1.6 Interactions and Interpersonal Relationships	
Describes how to perform the actions and behaviours that are necessary to establish basic and complex personal interactions with other people (strangers, friends, family, and lovers) in a way that is appropriate to the context and social environment.	Mental limitations that make it difficult for them to relate to others
1.7 Community, Social, and Civic Life	
Actions and tasks necessary to participate in organised social life outside the family, in areas of community, social, and civic life.	Needs help to participate in social and community life, needs help to get around outside the home, needs support for locomotion
2. Environmental Factors	
2.1 Products and Technologies	
Any product, instrument, piece of equipment, or technology adapted or designed specifically to improve the functioning of a person with a disability.	Needs supports for prosthesis use, needs supports for orthosis use, does not know how to use a PC, computer, tablet, or mobile device/smartphone
2.2 Support and Relationships	
People and animals that provide support to other people, both physical and emotional, as well as support in aspects of nutrition, protection, assistance and relationships, in their homes, in their workplaces, at school, at play, or in any other aspect of their daily activities.	From whom do they receive support to perform ADLs? Interest in using programmes of the Uruguayan Integrated Care System, and whether or not they feel lonely.
2.3 Services, Systems, and Policies	
“Services and the people who provide these services” [1] (p. 204), which represent the provision of benefits, structured programmes, and operations, which may be public, private, or voluntary, and developed at local, community, regional, state, provincial, national, or international levels, by employers, associations, organisations, agencies, or governments, in order to meet the needs of individuals.	Refusal of benefit claim linked to health condition; received sickness, accident or temporary disability benefit; received food support (food basket, card, canteen) in the last 12 months; in need of financial support

**Table 2:** Socio-demographic characteristics of the sample according to groups of constraints.

	More than One Limitation (n = 210)	Visual Limitations (n = 310)	Communication Limitations (n = 66)	Physical Limitations (n= 371)	Learning Limitations (n = 59)	Limitations in Relating to Others (n = 16)
Highest level of education attained * (n (%))	(*p* = 0.000)	(*p* = 0.002)	(*p* = 0.778)	(*p* = 0.062)	(*p* = 0.007)	(*p* = 0.235)
Incomplete First Grade Education	44 (21.0)	18 (5.8)	7 (10.6)	35 (9.4)	15 (25.4)	1 (6.3)
Complete First Grade Education	51 (24.3)	76 (24.5)	22 (33.3)	101 (27.2)	23 (39.0)	4 (25.0)
Incomplete Secondary Grade Education	49 (23.3)	93 (30.0)	14 (21.2)	97 (26.1)	8 (13.6)	7 (43.8)
Complete Secondary Grade Education	32 (15.2)	74 (23.9)	16 (24.2)	112 (30.2)	8 (13.6)	3 (18.8)
Incomplete Superior Education	11 (5.2)	28 (9.0)	3 (4.5)	12 (3.2)	1 (1.7)	1 (6.3)
Complete Superior Education	2 (1.0)	16 (5.2)	2 (3.0)	11 (3.0)	0 (0)	0 (0)
Working status (n (%) *p*)						
Did not work during the past week for at least one hour, excluding housework	161 (76.7) 0.000	139 (44.8) 0.000	36 (54.5) 0.263	242 (54.5) 0.263	38 (64.4) 0.568	14 (87.5) 0.029
Not available to begin work	124 (59.0) 0.164	75 (24.2) 0.001	16 (24.2) 0.001	181 (48.8) 0.002	28 (47.5) 0.974	8 (50.0) 0.699
Not looking for work during the past week	146 (69.5) 0.013	99 (31.9) 0.065	25 (37.9) 0.118	196 (52.8) 0.8	34 (57.6) 0.479	8 (50.0) 0.029

* The difference between the totals for each group and the disaggregated information by educational level is due to missing data from the original survey base.

**Table 3:** Diseases diagnosed according to groups of limitations (n (%) *p*).

	More than One Limitation (n = 210)	Visual Limitations (n = 310)	Communication Limitations (n = 66)	Physical Limitations (n= 371)	Learning Limitations (n = 59)	Limitations in Relating to Others (n = 16)
Reported diagnosed disease	56 (26.7) 0.008	33 (10.6) 0.000	9 (13.6) 0.173	89 (24.0) 0.070	19 (32.2) 0.017	5 (31.3) 0.265
Asthma	4 (7.1) 0.121	1 (3.0) 0.165	1 (11.1) 0.640	3 (3.4) 0.694	1 (5.3) 0.558	0 (0.0) 0.690
Arthritis/Arthrosis	10 (17.9) 0.457	4 (12.1) 0.005	1 (11.1) 0.305	25 (28.1) 0.000	0 (0.0) 0.000	0 (0.0) 0.000
High blood pressure	10 (17.9) 0.750	9 (27.3) 0.133	1 (11.1) 0.242	21 (23.6) 0.126	3 (15.8) 0.779	1 (20.0) 0.709
Renal insufficiency	1 (1.8) 0.048	0 (0.0) 0.513	0 (0.0) 0.794	0 (0.0) 0.454	0 (0.0) 0.806	0 (0.0) 0.000
Cardiological problems	2 (3.6) 0.655	5 (15.2) 0.505	0 (0.0) 0.343	6 (6.7) 0.441	0 (0.0) 0.372	0 (0.0) 0.000
Spinal problems	14 (25.0) 0.197	5 (15.2) 0.001	3 (33.3) 0.878	29 (32.6) 0.001	0 (0.0) 0.000	0 (0.0) 0.000
Cancer	1 (1.8) 0.822	0 (0.0) 0.000	0 (0.0) 0.000	4 (4.5) 0.203	1 (5.3) 0.247	0 (0.0) 0.000
Digestive/liver problems	0 (0.0) 0.000	0 (0.0) 0.000	0 (0.0) 0.000	2 (2.2) 0.642	0 (0.0) 0.000	0 (0.0) 0.000
Neurological disorders	10 (17.9) 0.001	2 (6.1) 0.048	1 (11.1) 0.797	4 (4.5) 0.134	2 (10.5) 0.405	1 (20.0) 0.208
Down syndrome	4 (7.1) 0.000	0 (0.0) 0.000	0 (0.0) 0.000	0 (0.0) 0.000	0 (0.0) 0.000	0 (0.0) 0.000
Parkinson’s	1 (1.8) 0.298	0 (0.0) 0.000	1 (11.1) 0.012	0 (0.0) 0.000	0 (0.0) 0.000	0 (0.0) 0.000
Autoimmune diseases	3 (5.4) 0.688	2 (6.1) 0.310	0 (0.0) 0.000	5 (5.6) 0.678	2 (10.5) 0.101	0 (0.0) 0.000
Epilepsy	3 (5.4) 0.567	1 (3.0) 0.430	0 (0.0) 0.000	3 (3.4) 0.547	4 (21.1) 0.000	0 (0.0) 0.676
Fibromyalgia	9 (16.1) 0.316	4 (12.1) 0.022	2 (22.2) 0.936	12 (13.5) 0.960	6 (31.6) 0.002	0 (0.0) 0.464
Obesity	2 (3.6) 0.858	1 (3.0) 0.128	0 (0.0) 0.384	8 (9.0) 0.011	0 (0.0) 0.412	0 (0.0) 0.676
Alcoholismo	0 (0.0) 0.312	1 (3.0) 0.826	0 (0.0) 0.601	2 (2.2) 0.558	1 (5.3) 0.096	0 (0.0) 0.802
Celiac disease	11 (19.6) 0.008	4 (12.1) 0.080	0 (0.0) 0.169	3 (3.4) 0.006	5 (26.3) 0.004	4 (80.0) 0.000
Allergies	1 (1.8) 0.822	1 (3.0) 0.474	0 (0.0) 0.521	4 (4.5) 0.116	0 (0.0) 0.546	0 (0.0) 0.758
HIV	0 (0.0) 0.475	2 (6.1) 0.031	0 (0.0) 0.712	0 (0.0) 0.289	0 (0.0) 0.728	0 (0.0) 0.859

## Data Availability

The database used can be requested at the following link: https://www.elps.org.uy/25/solicitar-base-de-datos.html. The authors signed an ethical safeguards agreement when working with the ELPS data at the time of requesting the database.

## Appendix A

**Table A1:** Contextual Factors described through the ELPS variables, by the type of limitation.

Contextual Factors (CIF)	Limitations Group (n = 1032)					
	More than One Limitation (n = 210)	Visual Limitations (n =310)	Communication Limitations (n = 66)	Physical Limitations (n =371)	Learning Limitations (n = 59)	Limitations in Relating to Others (n = 16)
1-Personal Factors						
1.1 LEARNING AND APPLICATION OF KNOWLEDGE						
They claim to have mental limitations that make it difficult for them to learn and apply knowledge and perform tasks (n (%) *p*)	109 (51.9) 0.000	0 (0) 0.000	0 (0) 0.000	0 (0) 0.000	59 (100) 0.000	0 (0) 0.000
This limitation does affect daily life	101 (92.7)	0 (0)	0 (0)	0 (0)	49 (83.0)	0 (0)
Cannot read or write (n (%) *p*)	43 (20.5) 0.000	4 (1.3) 0.000	4 (6.1) 0.857	3 (0.8) 0.000	14 (23.7) 0.000	0 (0) 0.000
Needs learning support (n (%) *p*)	56 (26.7) 0.000	7 (2.3) 0.000	1 (1.5) 0.029	1 (0.3) 0.000	26 (44.1) 0.000	1 (0.5) 0.707
Not receiving support	38 (67.9)	4 (57.1)	1 (100)	1 (100)	17 (65.4))	1 (100)
1.2 COMMUNICATION						
They claim to have a speech and communication limitations (n (%) *p*)	80 (38.1) 0.000	0 (0) 0.000	66 (100) 0.000	0 (0) 0.000	0 (0) 0.000	0 (0) 0.000
This limitation does affect daily life	20 (25.0)	0 (0)	36 (54.6)	0 (0)	0 (0)	0 (0)
They claim to need help to communicate and make decisions (n (%) *p*)	77 (36.7) 0.000	3 (1.0) 0.000	4 (6.1) 0.054	10 (2.7) 0.000	21 (35.6) 0.000	6 (2.9) 0.001
Require maximum substitution in order to perform the task	27 (35.1)	0 (0)	0 (0)	0 (0)	4 (19.0)	0 (0)
1.3 MOBILITY						
Declare to be limited in the use of upper limbs (arms and hands) (n (%) *p*)	85 (40.5) 0.000	0 (0) 0.000	0 (0) 0.000	185 (49.9) 0.000	0 (0) 0.000	0 (0) 0.000
This limitation does affect daily life	77 (90.6)	0 (0)	0 (0)	172 (93.0)	0 (0)	0 (0)
They claim to have a limitation in the use of lower limbs (legs and feet) (n (%) *p*)	107 (51.0) 0.000	0 (0) 0.000	0 (0) 0.000	279 (75.2) 0.000	0 (0) 0.000	0 (0) 0.000
This limitation does affect daily life	103 (96.3)	0 (0)	0 (0)	265 (95.0)	0 (0)	0 (0)
Declare the need for assistance to change and maintain body position (n (%) *p*)	61 29.0) 0.000	7 (2.3) 0.000	3 (4.5) 0.000	104 (28.0) 0.000	0 (0) 0.000	0 (0) 0.000
Require maximum substitution in order to perform the task	6 (9.8)	0 (0)	0 (0)	2 (1.9)	0 (0)	0 (0)
Report needing help to move around within the household (n (%) *p*)	47 (22.4) 0.000	2 (0.6) 0.000	1 (1.5) 0.008	69 (18.6) 0.000	2 (3.4) 0.040	0 (0) 0.000
Require maximum substitution in order to perform the task	5 (10.6)	0 (0)	0 (0)	3 (4.3)	0 (0)	0 (0)
Need support for housing adaptations (n (%) *p*)	28 (13.3) 0.000	3 (1.0) 0.000	1 (1.5) 0.004	31 (8.4) 0.032	1 (1.7) 0.012	0 (0) 0.000
Not receiving support	14 (50.0)	2 (66.7)	1 (100)	16 (51.6)	1 (100)	0 (0)
1.4 SELF-CARE						
Report needing help to avoid health risks. ask for help. or follow treatment (n (%) *p*)	150 (71.4) 0.000	6 (1.9) 0.000	3 (4.5) 0.025	29 (7.8) 0.027	8 (13.6) 0.477	2 (1.0) 0.806
Require maximum substitution in order to perform the task	15 (10.0)	1 (16.7)	1 (33.3)	8 (27.6)	0 (0)	0 (0)
Report needing help to get dressed (n (%) *p*)	55 (26.2) 0.000	2 (0.6) 0.000	1 (1.5) 0.004	76 (20.5) 0.000	2 (3.4) 0.021	1 (0.5) 0.265
Require maximum substitution in order to perform the task	10 (18.2)	0 (0)	0 (0)	5 (6.58)	0 (0)	0 (0)
Report needing help with combing hair. cutting nails. brushing hair. or brushing teeth (n (%))	56 (26.7) 0.000	5 (1.6) 0.000	1 (1.5) 0.006	62 (16.7) 0.001	3 (5.1) 0.016	0 (0) 0.000
Require maximum substitution in order to perform the task	20 (35.7)	3 (60.0)	0 (0)	7 (11.3)	0 (0)	0 (0)
No medical consultation in the last 12 months (n (%) *p*)	30 (14.3) 0.106	76 (24.5) 0.001	11 (16.7) 0.752	53 (14.3) 0.017	12 (20.3) 0.649	5 (31.3) 0.170
Papanicolau test was not performed (+) (n (%) *p*)	56 (49.1) 0.001	77 (36.2) 0.009	11 (30.6) 0.999	102 (40.3) 0.163	16 (44.4) 0.516	5 (83.3) 0.001
No mammography performed (^) (n (%) *p*)	37 (39.8) 0.178	59 (35.8) 0.133	15 (22.7) 0.908	83 (39.7) 0.143	9 (47.4) 0.020	3 (75.0) 0.024
No prostate screening (¬) (n (%) *p*)	44 (75.9) 0.452	57 (72.2) 0.444	15 (68.2) 0.265	59 (60.2) 0.042	13 (100) 0.016	7 (87.5) 0.752
1.5 DOMESTIC LIFE						
Report needing help with cooking. shopping. cleaning (n (%) *p*)	99 (47.1) 0.000	19 (6.1) 0.000	3 (4.5) 0.000	162 (43.7) 0.000	8 (13.6) 0.002	2 (1.0) 0.081
Require maximum substitution in order to perform the task	30 (30.3)	2 (10.5)	1 (33.3)	37 (22.8)	0 (0)	1 (50.0)
Need support for daily tasks (n (%) *p*)	66 (31.4) 0.000	7 (2.3) 0.000	1 (1.5) 0.001	83 (22.4) 0.000	11 (18.6) 0.644	2 (1.0) 0.666
Not receiving support	21 (31.8)	2 (28.6)	0 (0)	28 (33.7)	3 (27.3)	0 (0)
1.6 INTERACTIONS AND INTERPERSONAL RELATIONSHIPS						
Report having a mental limitation that makes it difficult for them to relate to others (n (%) *p*)	70 (33.3) 0.000	0 (0) 0.000	0 (0) 0.011	0 (0) 0.000	0 (0) 0.017	16 (100) 0.000
This limitation does affect daily life	66 (94.3)	0 (0)	0 (0)	0 (0)	0 (0)	15 (93.8)
1.7 COMMUNITY. SOCIAL. AND CIVIC LIFE						
Need support for locomotion (n (%) *p*)	53 (25.2) 0.000	8 (2.6) 0.000	0 (0) 0.001	67 (18.1) 0.000	4 (6.8) 0.150	1 (0.5) 0.290
Not receiving support	36 (67.9)	4 (50.0)	0 (0)	37 (55.2)	4 (100)	1 (100)
Report needing help to participate in social and community life (n (%) *p*)	84 (40.0) 0.000	14 (4.5) 0.000	3 (4.5) 0.004	62 (16.7) 0.621	12 (20.3) 0.544	5 (2.4) 0.145
Require maximum substitution in order to perform the task	18 (21.4)	1 (7.1)	1 (33.3)	3 (4.8)	1 (8.3)	0 (0)
Report needing help to move around outside the home (n (%) *p*)	100 (47.6) 0.000	20 (6.5) 0.000	2 (3.0) 0.000	131 (35.3) 0.000	8 (13.6) 0.010	2 (1.0) 0.142
Require maximum substitution in order to perform the task	10 (10.0)	0 (0)	0 (0)	8 (6.1)	0 (0)	0 (0)
2.1 PRODUCTS AND TECHNOLOGIES						
Needs support for prostheses (n (%) *p*)	11 (5.2) 0.045	2 (0.6) 0.003	1 (1.5) 0.302	18 (4.9) 0.015	0 (0) 0.000	0 (0) 0.471
Not receiving support	9 (81.8)	1 (50.0)	0 (0)	9 (50.0)	0 (0)	0 (0)
Need supports for orthoses (n (%) *p*)	55 (26.2) 0.000	39 (12.6) 0.291	8 (12.1) 0.595	45 (12.1) 0.129	1 (1.7) 0.004	0 (0) 0.000
Not receiving support	37 (67.3)	22 (56.4)	6 (75.0)	26 (57.8)	1 (100)	0 (0)
Do not know how to use a PC or computer or tablet or mobile device smartphone	77 (36.7) 0.144	106 (34.2) 0.017	28 (42.4) 0.463	147 (39.6) 0.911	22 (37.3) 0.216	9 (4.3) 0.183
Did not use a PC or computer or tablet or smartphone mobile device in the last 30 days (n (%) *p*)	13 (6.2) 0.097	15 (4.8) 0.274	3 (4.5) 0.882	20 (5.4) 0.861	3 (5.1) 0.526	0 (0) 0.008
2.2 SUPPORT AND RELATIONSHIPS						
Is assisted by another person in making ADLs	135 (64.3) 0.004	30 (9.7) 0.002	4 (6.1) 0.232	178 (48.0) 0.273	28 (47.5) 0.798	4 (25.0) 0.543
Unpaid caregiver	122 (90.4)	22 (73.3)	2 (50.0)	155 (87.1)	24 (85.7)	4 (100)
Paid caregiver	6 (4.4)	0 (0)	1 (25.0)	3 (1.7)	0 (0)	0 (0)
Institution	1 (0.7)	0 (0)	0 (0)	0 (0)	1 (3.6)	0 (0)
Does not receive help	4 (3.0)	7 (23.3)	1 (25.0)	18 (10.1)	2 (7.1)	0 (0)
Interested in Care Programme (*¬)	(*p* = 0.008)	(*p* = 0.300)	(*p* = 0.790)	(*p* = 0.096)	(*p* = 0.390)	(*p* = 0.045)
Yes	66 (31.4)	70 (22.6)	15 (22.7)	81 (21.8)	21 (35.6)	1 (6.3)
No	64 (30.5)	154 (49.7)	16 (24.2)	154 (41.5)	14 (23.7)	5 (31.3)
Do not know about the subject	73 (34.8)	81 (26.1)	33 (50.0)	132 (35.6)	22 (37.3)	10 (62.5)
Feelings of loneliness	98 (46.7) 0.000	73 (23.5) 0.000	21 (31.8) 0.462	131 (35.3) 0.563	35 (59.3) 0.000	12 (75.0) 0.001
2.3 SERVICES. SYSTEMS. AND POLICIES						
Have been refused a benefit claim related to their health condition (n (%) *p*)	45 (21.4) 0.000	15 (4.8) 0.000	7 (10.6) 0.583	53 (14.3) 0.282	9 (15.3) 0.560	3 (1.4) 0.472
Received food support (basket. card. canteen) in the last 12 months (n (%) *p*)	30 (14.3) 0.216	26 (8.4) 0.000	9 (13.6) 0.637	42 (11.3) 0.709	13 (22.0) 0.012	2 (1.0) 0.933
Received sickness. accident. or temporary disability benefit (n (%) *p*)	23 (11.0) 0.697	25 (8.1) 0.017	3 (4.5) 0.008	65 (17.5) 0.000	3 (5.1) 0.103	2 (1.0) 0.923
In need of financial support (n (%) *p*)	101 (48.1) 0.000	32 (10.3) 0.000	12 (18.2) 0.091	104 (28.0) 0.626	25 (42.4) 0.019	6 (2.9) 0.348
Not receiving support	68 (67.3)	24 (75.0)	8 (66.7)	88 (84.6)	16 (64.0)	5 (83.3)

(+) Calculated based on the total number of women in the study sample. (^) Calculated based on the total number of women over 40 in the study sample. (¬) Calculated based on the total number of men over 40 years old in the study sample. (*¬) Calculated on the basis of the total number of people who reported needing help from another person to perform ADLs.
